# ERG Protein Expression Is of Limited Prognostic Value in Men with Localized Prostate Cancer

**DOI:** 10.1155/2013/786545

**Published:** 2013-08-19

**Authors:** Liang Hong Teng, Cheng Wang, Michael Dolph, Bryan Donnelly, Tarek A. Bismar

**Affiliations:** ^1^Department of Pathology and Laboratory Medicine, University of Calgary and Calgary Laboratory Services, Calgary, AB, Canada T2V 1P9; ^2^Department of Urology, University of Calgary, Calgary, AB, Canada T2N 1N4; ^3^The Prostate Cancer Center, Calgary, AB, Canada T2V 1P9; ^4^Department of Oncology, Biochemistry and Molecular Biology, Calgary, AB, Canada T2N 1N4; ^5^Southern Alberta Cancer Institute and Tom Baker Cancer Center, Calgary, AB, Canada T2N 1N4

## Abstract

*Background*. The prognostic significance of ERG expression in prostate cancer (PCA) has generated mixed results. We sought to investigate the prognostic significance of ERG expression in a localized cohort of men with PCA. *Material and Methods*. We investigated ERG protein expression in a cohort of 198 men with localized PCA. ERG expression was correlated with patients' clinical outcome and several pathological parameters, including Gleason score (GS), pathological stage, surgical margin, and extra-capsular extension. *Results*. ERG expression was detected in 86/198 (43.4%) patients exclusively in neoplastic epithelium. Overall, ERG mean expression intensity was 1.01 ± 1.27 versus 0.37 ± 0.83 in acinar PCA compared to foamy type PCA (*P* < 0.001). In HGPIN, ERG intensity levels were comparable to those in foamy type PCA (0.13 ± 0.56) but significantly lower than those in acinar PCA (*P* < 0.001). ERG expression was significantly associated with extra-prostatic extension and higher pathological stage and showed a trend toward seminal vesicle invasion. Herein, ERG expression was documented in 50/131 (38.1%) patients with pT2 versus 30/55 (54.5%) patients with pT3 (*P* = 0.04). ERG association with higher pathological stage was more pronounced in patients with GS > 7. Grouping patients into those with GS ≤ 7 versus >7, there was no significant association between ERG expression and GS. Similarly, no association was present in relation to either surgical margins or postsurgical serum PSA levels. *Conclusion*. We report significant association between ERG protein levels and extra-prostatic extension and higher pathological stage. ERG expression is not associated with adverse clinical outcome and is of limited prognostic value in localized PCA.

## 1. Introduction

ERG protein expression has been recently suggested to be reflective of *ERG* gene rearrangements in prostate cancer (PCA) documenting remarkable concordance between the two [1–6]. The rearrangements between the androgen receptor-regulated gene *TMPRSS*2 (21q22.3) and members of the *ETS* family member of transcription factor gene, most commonly ERG (21q22.2), are among the most common genetic alterations detected in prostate cancer [7–11]. *ERG* gene rearrangements have been detected in roughly half (40–60%) of PCA of surgical cohorts compared to a rate of 12%–15% in incidental or watchful waiting cohorts [7, 12–18].

Previous studies investigating the prognostic significance of *ERG* gene rearrangements have revealed mixed results [19–22]. However, it is becoming more evident that *ERG* gene rearrangements signify a molecular subtype of PCA.

Some studies investigating the significance of ERG protein expression in localized PCA failed to show association with adverse clinical outcome [23, 24]. However, a recent report by our group demonstrated an association of ERG expression with lethal disease in patients with unsuspected and advanced/castrate resistant disease who were treated by transurethral resection of prostate [25]. Moreover, we documented significant association between ERG expression and both Gleason score and tumor volume. Studies from our group and others have also linked ERG status to responsiveness to hormonal therapy, and longer progression time to castration resistant disease, compared to men with no ERG expression [24, 25]. In the current study, we investigated the association of ERG protein expression to clinical-pathological parameters in a cohort of men with localized prostate cancer.

## 2. Material and Methods

### 2.1. Study Population and Tissue Microarray Construction

The study cohort consisted of 198 patients who were treated by retropubic radical prostatectomy for localized prostate cancer with a mean followup of 4.8 years (range 0–15.8). Clinical and pathological data were obtained with approval from the institutional review board. Clinical progression was defined as a postoperative serum PSA elevation of >0.2 ng/mL. Prostate samples were embedded onto three tissue microarray (TMA) blocks using a manual tissue arrayer (Beecher Instruments, Silver Spring, MD, USA). Each block was assembled without prior knowledge of any clinical or pathological staging information. One to nine cores (average 3.3), 0.6 mm in diameter, were sampled including benign, high grade intraepithelial neoplasia (HGPIN), and prostate cancer (PCA). After construction, 4 *μ*m sections were cut and stained with haematoxylin and eosin on the initial slides to verify the histological diagnosis. The study patients' demographics have been previously described [26, 27].

### 2.2. ERG Protein Expression by Immunohistochemistry (IHC)

Briefly, 4 *μ*m thick sections from formalin-fixed paraffin-embedded tissue blocks were stained with Ventana autostainer. Prior to the staining, heat-induced antigen retrieval was carried out by vegetable steamer in sodium citrate antigen retrieval buffer (10 mM pH 6.0) for 40 minutes and then cooled down to room temperature for about 20 minutes. The slides were incubated for 60 minutes at 37°C with ERG rabbit monoclonal antibody (Epitomics, clone EPR 3864) at 1 : 50 dilution. A Ventana iView DAB detection kit (Ventana Tucson, AZ, USA) was used for HR detection and counter stain.

### 2.3. Pathological Analysis

The diagnoses of all TMA cores were confirmed by the three study pathologists (LHT, CW, and TAB). Gleason scoring was done according to the 2005 ISUP criteria [28]. For each patient, the two predominant patterns were sampled and included on the TMAs for analysis. ERG protein expression was assessed semiquantitatively using 3-tiered system (0, negative; 1, low; 2, high). Cases with either 1 or 2 intensity were considered positive based on previous correlation with *ERG *gene rearrangement as detected by fluorescent in situ hybridization (data not shown). The ERG antibody was consistently strongly expressed in endothelial cells, which acted as internal control for expression and intensity level.

### 2.4. Statistical Analysis

Patient characteristics were presented as frequencies and percentages for categorical variables and as means and ranges for continuous variables. Chi-square tests were used to test for associations between ERG protein expression and Gleason score, surgical margin, and pathological stage. The Kaplan-Meier approach along with the log-rank test was used for the survival analyses to test the association between ERG expression and serum PSA relapse. In all statistical tests, a *P* value <0.05 was considered significant.

## 3. Results

### 3.1. Expression of ERG Protein by Anti-ERG Monoclonal Antibody in Benign, HGPIN, and PCA

**Figure 1:**
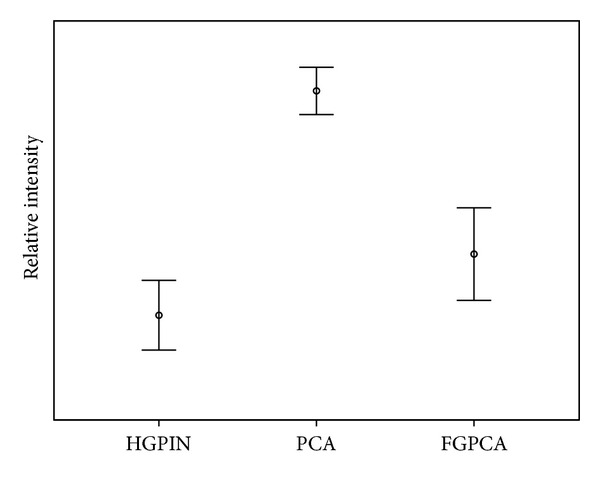
Mean intensity level of ERG expression in high grade prostatic intraepithelial neoplasia (HGPIN), foamy gland type prostate cancer (FGPCA), and acinar prostate cancer (PCA). Error bars represent 95% CI of mean ERG intensity.

**Figure 2:**
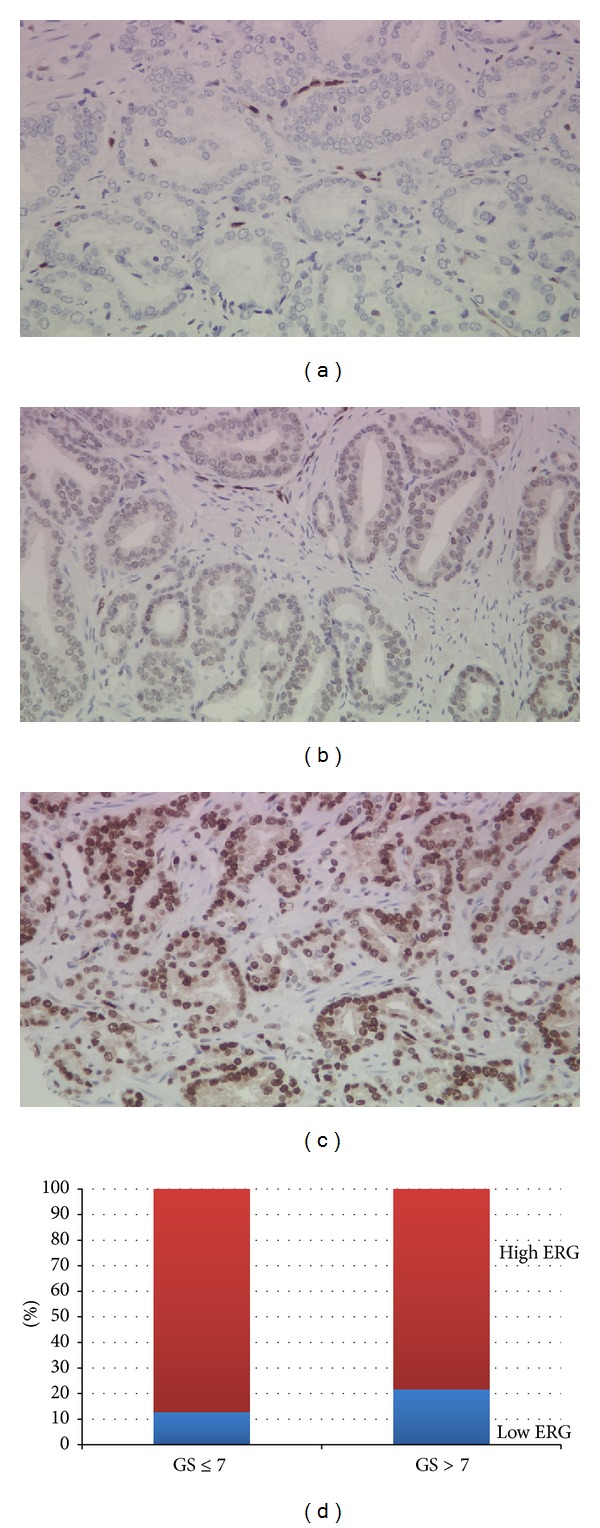
(a) Prostate cancer Gleason score 6, negative for ERG expression. Note that endothelial cells acting as positive control show strong ERG expression. (b) Prostate cancer Gleason score 6, showing weak intensity ERG expression. (c) Prostate cancer Gleason score 7 (3 + 4), showing high intensity ERG expression. (d) ERG low and high intensity distribution percentages in patients with GS ≤ 7 versus >7.

Mean patients' age of this cohort was 64 years (range 42.7–80.5 years) with average follow-up time of 4.8 years (range 0.0–15.8 months). ERG protein expression was detected in 86/198 patients (43.4%).  Table 1 demonstrates patients' demographics of the study cohort with respect to ERG expression. Overall, there were no significant differences between the two subgroups (ERG pos & ERG neg) of patients except for pathological stage, with 37% of ERG positive tumors detected in pT3 versus 24% in pT2. To investigate ERG expression in different diagnostic categories, we characterized ERG expression based on individual cores sampled. ERG protein expression was detected in 317/788 (40.2%) PCA cores. The rate of ERG expression in HGPIN was 4/69 (5.8%). When we accounted for foamy type PCA morphology, the rate of ERG expression was 15/84 (17.9%), compared to 302/704 (42.9%) in acinar PCA (cases with no foamy type morphology) (*P* < 0.001). There was no difference in high ERG intensity between foamy type and acinar PCA (data not shown). However, mean intensity level of ERG in acinar PCA was significantly higher than foamy type PCA, 1.01 ± 1.27 versus 0.37 ± 0.83 (*P* < 0.001). ERG intensity levels in HGPIN were comparable to those in foamy type PCA but slightly lower (0.13 ± 0.56) but significantly lower than those in acinar PCA (*P* < 0.001) (Figure 1). There were no significant differences in ERG expression between different Gleason scores. ERG expression was noted in 106/280 (37.8%), 175/463 (37.8%), and 37/108 (34.2%) of Gleason scores 6, 7, and 8–10, respectively.  Figure 2 demonstrates examples of ERG expression in tissue samples and the distribution of ERG in relation to Gleason score.

### 3.2. Expression of ERG in relation to Clinical-Pathological Parameters and Postsurgical Serum PSA Relapse

**Figure 3:**
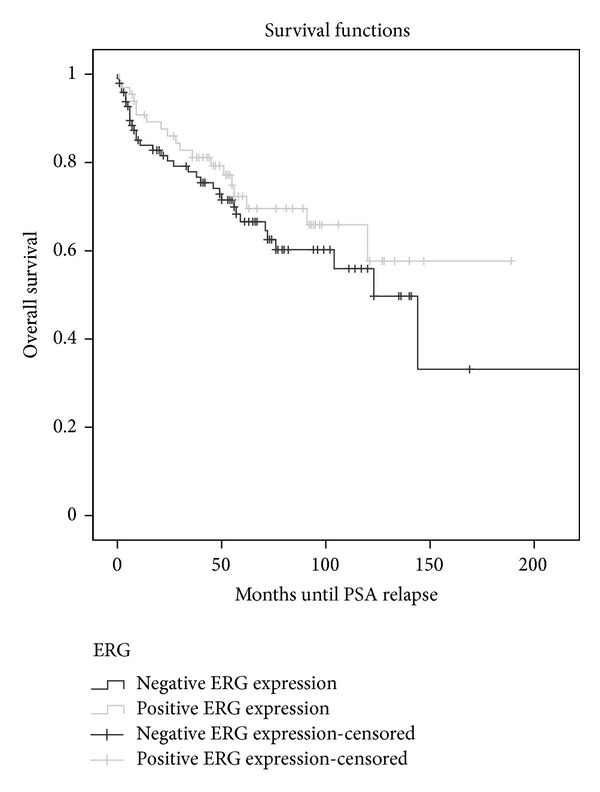
Kaplan-Meier curve of association between ERG expression and PSA relapse after radical prostatectomy (*P* = 0.31).

When investigating relations between ERG expression and pathological parameters, there was significant association between ERG expression and higher disease stage. In this cohort, ERG expression was present in 50/131 (38.1%) patients with pT2 versus 30/55 (54.5%) patients with pT3 (*P* = 0.04). A similar association was also noted between ERG expression and extra-capsular extension. In this cohort, 52/134 (38.8%) ERG positive patients demonstrated organ confined disease versus 29/53 (54.7%) ERG positive patients showing extra-capsular extension (*P* = 0.04). Similar trends were noted between ERG expression and seminal vesicle invasion, but this was not statistically significant (*P* = 0.10) (data not shown). No significant association was noted between overall ERG positivity and positive surgical margins (Table 1). Similarly, no association was observed with postsurgical PSA levels when assessed by univariate or multivariate analysis (Figure 3).

Although not informative due to limited patients' numbers, the association between ERG expression and higher stage disease was more pronounced in patients with higher GS. In this cohort, none of the patients with GS > 7 and ERG positive (0/7) were of stage pT2 compared to 47% (7/15) of ERG negative patients who were of pT2 stage (*P* = 0.02) (Table 2).

## 4. Discussion

This study reports on the potential significance of ERG protein expression in localized prostate cancer. *ERG *gene rearrangements and ERG expression have been documented in roughly 50% of localized prostate and locally advanced castrate resistant prostate cancer compared to 12%–15% in watchful waiting or incidental cohorts [16, 17, 22, 25, 29]. Published reports on the significance of ERG expression to patients' outcome are conflicting with some showing association with adverse outcome, while others document no association. Some suggest that it indicates a better prognosis [16, 22, 25, 29–33]. This question still needs confirmation in larger studies. However, it is proposed that ERG signifies a molecular class of prostate cancer and may play a role in disease progression within those tumors. This pathway is closely linked to increased rate of *PTEN* genomic deletions as well as increased ERG expression or *ERG *gene rearrangements [34, 35]. *ERG *gene rearrangements and ERG expression are associated with adverse outcome and lethal disease in watchful waiting or expectant cohorts [16, 25]. Moreover, patients with ERG overexpression demonstrated shorter progression times to castrate resistance, needing surgical intervention (channel TURP) [25]. In localized prostate cancer, the majority of the reported data suggest no prognostic implication for *ERG *rearrangements or ERG overexpression in relation to PSA relapse or the recurrence of local disease [22, 36, 37]. However, two earlier reports suggested adverse association of *ERG *rearrangements with PSA relapse after radical prostatectomy [20, 31]. In Nam's study, ERG status was evaluated using PCR and direct sequencing. Yashimoto's group noted that the adverse prognostic association was linked to *PTEN* genomic deletions. Of note, a study by Reid et al. documented that *ERG *gene rearrangements in addition to *PTEN *genomic deletions had actually favorable outcomes compared to *PTEN* deletion alone [19]. These observations suggest that the method utilized in determining ERG status, the inclusion or exclusion of other genomic aberration, and the cohort chosen may be the reason for the different results obtained in various studies investigating the role of ERG in prostate cancer. Our results support previous observations showing no prognostic relationship between ERG overexpression and clinical outcome in localized prostate cancer. However, in contrast to earlier studies, we document significant association between ERG expression and some pathological parameters. In this cohort, patients whose tumors were ERG positive were at higher risk of exhibiting extra-prostatic extension and increased disease stage compared to patients whose tumors did not express ERG. Specifically, all patients with GS > 7 and ERG expression showed pT3 stage disease compared to none of pT2. However, this data is limited by the number of patients within each GS subgroup and need further confirmation. More importantly, in addition to not documenting any clinical prognostic significance for ERG expression, we did not observe any association between ERG expression and other pathological parameters such as Gleason score or surgical margins, which further diminishes any potential prognostic significance for ERG expression, at least in localized PCA.

Another issue worth mentioning regarding *ERG* is that,* ERG *gene rearrangements have been previously associated with specific histopathological features and detected more frequently in some morphologic variants of prostate cancer than others [38]. In this study, we confirm lower rates of ERG expression in tumors with foamy/xanthomatous morphology which demonstrated lower mean expression intensities compared to acinar PCA. The significance of this is not yet known, but suggests that those two types of tumors may be different at the molecular level.

In conclusion, this study demonstrates significant association between ERG expression and extra-prostatic extension and higher pathological stage in localized prostate cancer. It also demonstrates no prognostic correlation of ERG expression with patients' clinical outcome. Moreover, the lack of association with any other pathological parameters also significantly diminishes any potential clinical application of ERG expression, at least in men with localized prostate cancer.

## Figures and Tables

**Table 1:** Demographics of the study patients' cohort.

	ERG negative (112 patients)	ERG positive (86 patients)	*P* value
Age (years) (mean; range)	64.31 (43–81)	64.00 (47–75)	0.735
Pre-PSA level (ng/mL)			0.652
≤10	50 (69%)	33 (73%)	
>10	22 (31%)	12 (27%)	
Gleason summary			0.521
<7	34 (31%)	30 (35%)	
7	61 (55%)	47 (55%)	
3 + 4	36 (32%)	27 (31%)	
4 + 3	25 (23%)	20 (24%)	
>7	16 (14%)	8 (10%)	
pT stage			0.04
pT2	81 (76%)	50 (63%)	
pT3	25 (24%)	30 (37%)	
Surgical margin			0.886
Negative	60 (57%)	45 (56%)	
Positive	46 (43%)	36 (44%)	

**Table 2:** ERG expression in relation to pathological stage in Gleason score subgroups.

ERG expression	Path stage		*P* value
	pT2	pT3	
GS > 7			
ERG-Neg	7 (100%)	8 (53%)	*P* (0.02)
ERG-Pos	0 (0%)	7 (47%)	

Total	7	15	

GS < 7			
ERG-Neg	27 (56%)	2 (22%)	*P* (0.06)
ERG-Pos	21 (44%)	7 (78%)	

Total	48	9	
